# Association between haptoglobin polymorphism and coronary artery disease: a meta-analysis

**DOI:** 10.3389/fgene.2024.1434975

**Published:** 2024-09-11

**Authors:** Jiao Wang, Xiaokai Zhou, Yue Su, Dongjian Chai, Yaoyao Ruan, Jinhua Wang

**Affiliations:** Department of Cardiology, The Quzhou Affiliated Hospital of Wenzhou Medical University (Quzhou People’s Hospital), Quzhou, Zhejiang, China

**Keywords:** coronary artery disease, haptoglobin, polymorphism, meta-analysis, susceptibility

## Abstract

**Background:**

Previous studies have investigated the association between the haptoglobin rs72294371 polymorphism and coronary artery disease (CAD) risk, but the results are controversial and uncertain. Therefore, this study aimed to systematically review the literature on haptoglobin polymorphism and susceptibility to CAD.

**Methods:**

PubMed, Embase, Web of Science, Cochrane Library, and Wanfang databases were used to identify relevant studies from their inception to April 2024. The pooled odds ratio (OR) with corresponding 95% confidence interval (CI) were used to assess the strength of the association. An OR value greater than one suggested an increased risk; otherwise, it suggested a protective risk.

**Results:**

A total of 15 studies comprising 8,632 individuals (2,988 cases and 5,644 controls) were included. In the current meta-analysis, a significant association between haptoglobin polymorphism and CAD was found under recessive model (OR:0.74, 95% CI:0.60–0.92), dominant model (OR: 0.82, 95% CI: 0.71–0.95), homozygote model (OR: 0.70, 95% CI: 0.53–0.92), and allelic genetic model (OR: 0.80, 95% CI: 0.69–0.94). In the analysis stratified by ethnicity, a statistically significant association was observed in Asians rather than Caucasian population.

**Conclusion:**

This meta-analysis indicates that haptoglobin polymorphism is associated with CAD susceptibility, especially in Asians.

## Introduction

Coronary artery disease (CAD) is the leading cause of death worldwide, accounting for 30% of global mortality (Erdmann et al., 2018). The main cause of CAD is currently believed to be the accumulation of atherosclerotic plaques in blood vessels and subsequent blockage, which leads to cardiac hypoxia and nutrient deprivation (Moras et al., 2024). Despite substantial advancements in diagnosing and predicting CAD outcomes, the exact mechanisms underlying CAD onset remain elusive. Epidemiological studies have shown that the development of CAD is influenced by multiple factors, such as hypertension, smoking, diabetes, and dyslipidaemia (Gray et al., 2024). In addition, recent evidence suggests that environmental factors and genetic polymorphisms play a key role in the development of CAD (Charmet et al., 2018).

Haptoglobin, which is predominantly synthesized in the liver, is a glycoprotein with antioxidant properties (Orchard et al., 2016). It stabilizes free haemoglobin by binding to it, thereby preventing further tissue damage in inflamed regions (Dalan and Liuh Ling, 2018). The gene encoding haptoglobin is located at chromosome 16q22.3 and exists in two allelic forms, HP1 and HP2, which result in three genotypes, HP1-1, HP2-1, and HP2-2 (Dalan and Liuh Ling, 2018). Proteins derived from these genotypes exhibit distinct functional profiles; specifically, the HP2-2 phenotype, compared to the HP1-1 and HP2-1 phenotypes, exhibits compromised antioxidant capacity, potentially enhancing vulnerability to atherosclerosis and increasing the risk of CAD (Cahill et al., 2024; Warren et al., 2024). Although multiple studies have investigated the association between haptoglobin polymorphisms and CAD risk (Levy et al., 2004; Fan et al., 2013; Mewborn et al., 2024; Moras et al., 2024), a clear consensus among these studies has not been reached due to the small sample sizes and the limitations of research heterogeneity. Thus, this meta-analysis aimed to investigate the relationship between haptoglobin polymorphisms and CAD susceptibility by synthesizing findings from published research.

## Materials and methods

The present meta-analysis adheres to the Preferred Reporting Items for Systematic reviews and Meta-Analyses (PRISMA) statement (Page et al., 2021). The PRISMA Checklist was described in Supplementary Table S1.

### Search strategy

PubMed, Embase, Web of Science, Cochrane Library, and Wanfang databases were used to identify relevant studies from their inception to April 2024. The following keywords were used: haptoglobin, polymorphism, variant, mutation, coronary artery disease, and coronary heart disease. To avoid missing any potential study, the references of the included studies were also manually searched. Languages were limited to English and Chinese. The detailed search strategy was described in Supplementary Table S2.

### Inclusion and exclusion criteria

The eligible studies should match the inclusion criteria: 1) case-control study; 2) evaluate the association between haptoglobin polymorphism and the risk of CAD; 3) report sufficient data to calculate odds ratio (OR) and corresponding 95% confidence interval; 4) control population was consistent with Hardy–Weinberg equilibrium (HWE). The exclusion criteria were as follows: abstracts, case reports, reviews, duplicated studies, or incomplete data.

### Data extraction and quality assessment

Two investigators completed the data extraction independently, and any disagreement was discussed and resolved with consensus. The following information was extracted from eligible studies: the first author’s name, published year, country of origin, ethnicity, sample size, frequencies of genotypes in cases and controls, and Hardy-Weinberg equilibrium (HWE) in controls. The quality of the selected studies was assessed using the Newcastle–Ottawa Scale (NOS) (Stang, 2010).

### Statistical analysis

Statistical analyses were performed with STATA version 12.0 software (STATA Corp., College Station, TX, United States). Odds ratios (OR) with 95% confidence intervals (CI) were assessed to determine the relationship between the haptoglobin polymorphism and the risk of CAD under dominant (Hp 2-1 + Hp 2-2 vs. Hp 1-1), recessive (Hp 2-2 vs. Hp 1-1 + Hp 2-1), allelic (Hp 2 vs. Hp 1), codominant homozygote (Hp 2-2 vs. Hp 1-1), codominant heterozygote (Hp 2-1 vs. Hp 1-1), and overdominance genetic models (Hp 2-1 vs. Hp 1-1 + Hp 2-2). The HWE of the included studies was assessed by a chi-square test. The heterogeneity among the included studies was evaluated through the chi-squared test and I^2^ statistic. A fixed effect model (Mantel–Haenszel) was employed when I^2^ < 50%; otherwise, a random effect model (DerSimonian–Laird) was adopted. Stratified analyses were performed based on ethnicity. A sensitivity test was performed to evaluate the stability of the results. The publication bias was assessed by using Begg’s funnel plot and Egger’s linear regression test. A *P*-value <0.05 was considered statistically significant.

## Results

### Literature search and study characteristics

**FIGURE 1:**
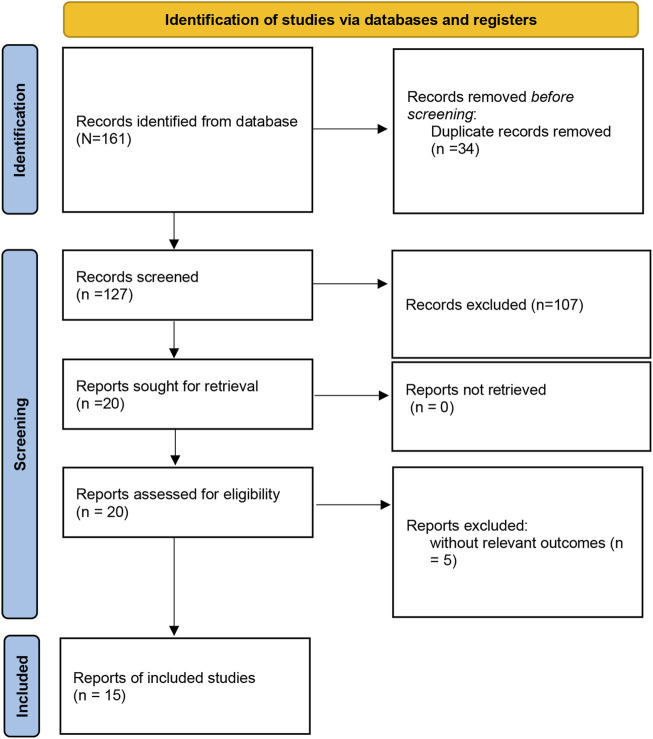
Flow chart of the eligible studies included in this meta-analysis.

The study selection process was presented in Figure 1. A total of 161 studies were acquired from PubMed, Embase, Web of Science, Cochrane Library, and Wan Fang databases. Among them, 34 articles were excluded as duplicates, 107 studies were excluded based on titles and abstracts, and 20 articles were excluded after reading the full text. Ultimately, a total of 15 articles (Bilgrami et al., 1980; Surya Prabha et al., 1987; Hong et al., 1997; Levy et al., 2004; Liu et al., 2011; Wobeto et al., 2011; Cahill et al., 2013; Fan et al., 2013; Lee et al., 2013; Pan et al., 2013; Hamdy et al., 2014; Moussa et al., 2014; Cahill et al., 2015; Wang et al., 2018; Mewborn et al., 2024) involving 2,988 cases and 5,644 controls were selected for this meta-analysis. The characteristics of the included studies were listed in Table 1. Studies were conducted between 1979 and 2024. Four studies involved America, 5 studies involved China, 2 studies involved India, and 1 study involved Korea, Brazil, Tunisia, and Egypt. The 4 studies were performed among Caucasians, 8 studies among Asians, 1 among Egyptian, Tunisian, and mixed population. The quality of the selected studies was assessed in Supplementary Table S3.

**TABLE 1:** The main characteristics of the included studies.

First author	Year	Country	Ethnicity	Sample size		Case			Control			HWE (Control)	NOS score
				Case	Control	Hp 1–1	Hp 2–1	Hp 2–2	Hp 1–1	Hp 2–1	Hp 2–2		
Bilgram	1979	India	Asian	40	50	2	8	30	2	8(	40	0.086	6
Prabha	1987	India	Asian	45	180	0	5	40	6	40	134	0.176	6
Seung	1997	Korea	Asian	220	347	15	79	126	23	141	183	0.550	6
Levy	2003	America	Caucasian	297	2,976	39	145	113	449	1,403	1,124	0.744	7
Liu	2011	China	Asian	189	242	13	74	102	31	126	85	0.136	6
Wobeto	2011	Brazil	Mixed	50	70	14	23	13	15	34	21	0.858	8
Cahill	2013	America	Caucasian	407	411	58	188	161	66	189	156	0.491	8
Lee	2013	China	Asian	359	83	27	145	187	6	35	42	0.724	6
Fan	2013	China	Asian	47	74	9	18	20	8	26	40	0.243	6
Pan	2013	China	Asian	98	77	19	25	54	20	30	27	0.060	7
Hamdy	2014	Egypt	Egyptian	48	72	6	12	30	14	37	21	0.750	6
Moussa	2014	Tunisia	Tunisian	256	144	56	121	79	42	74	28	0.652	7
Cahill	2016	America	Caucasian	695	696	112	317	266	109	312	275	0.193	8
Wang	2018	China	Asian	165	116	31	87	47	39	53	24	0.447	6
Mewborn	2024	America	Caucasian	72	106	6	40	26	23	58	25	0.329	8

Hp, Haptoglobin; HWE, Hardy–Weinberg equilibrium.

### Meta-analysis results for haptoglobin polymorphism

**FIGURE 2:**
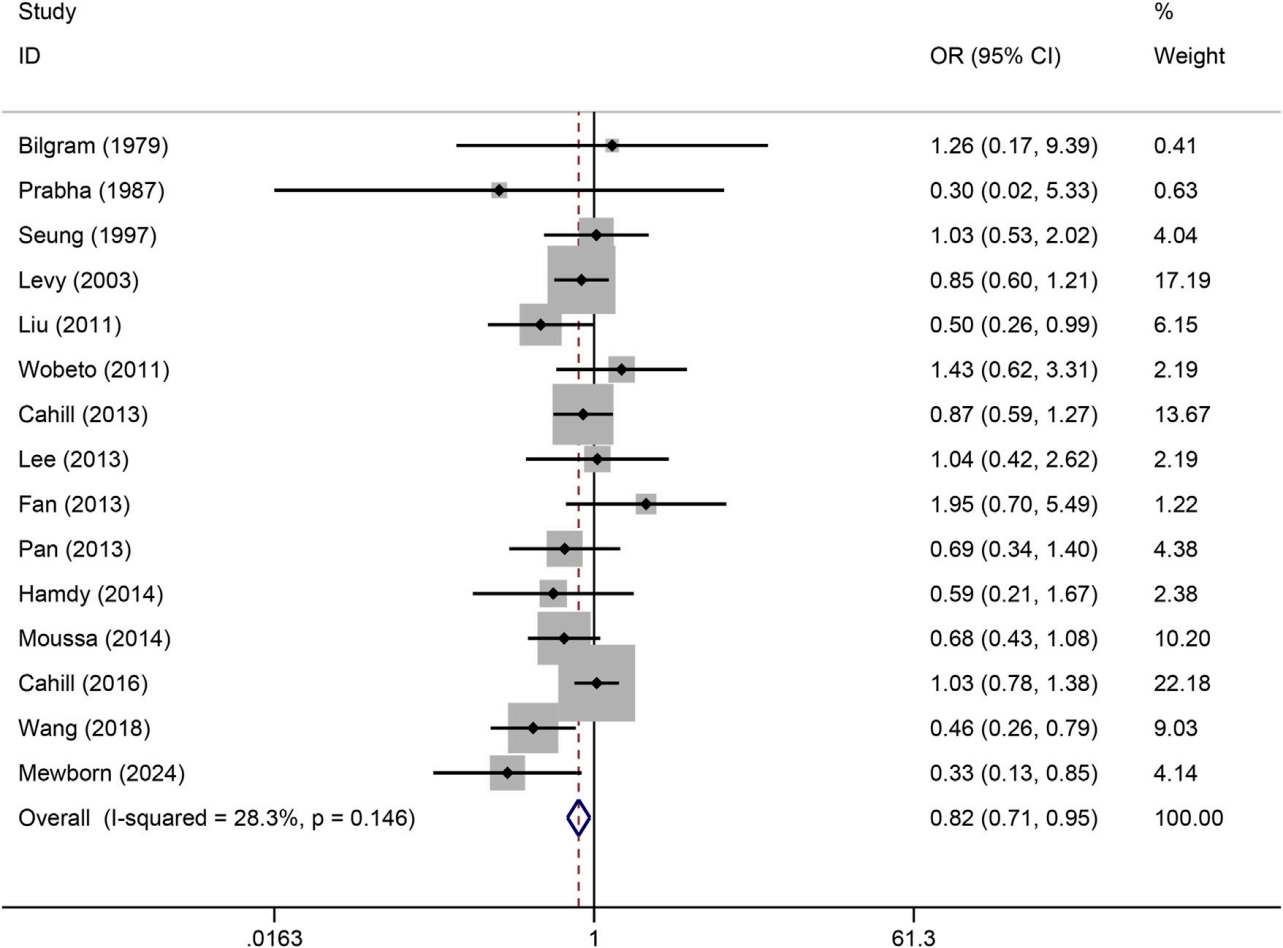
Forest plots of haptoglobin polymorphism and CAD risk under dominant genetic model.

**FIGURE 3:**
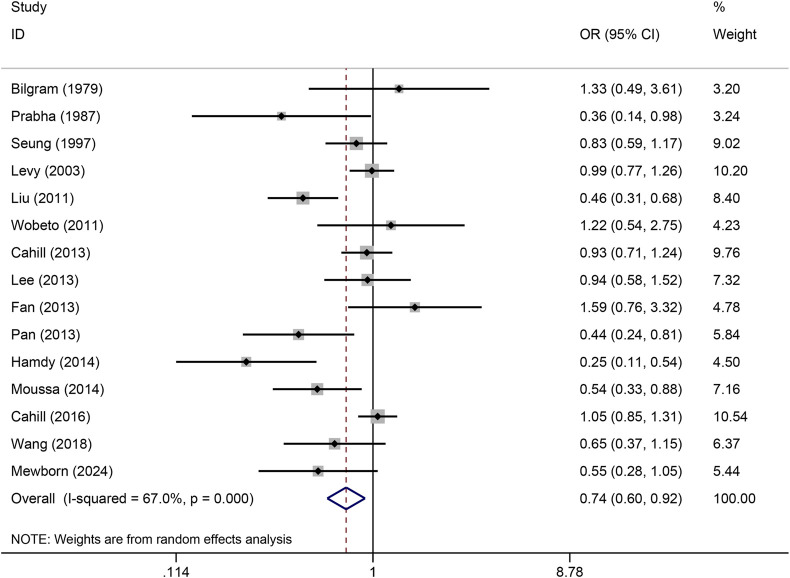
Forest plots of haptoglobin polymorphism and CAD risk under recessive genetic model.

**FIGURE 4:**
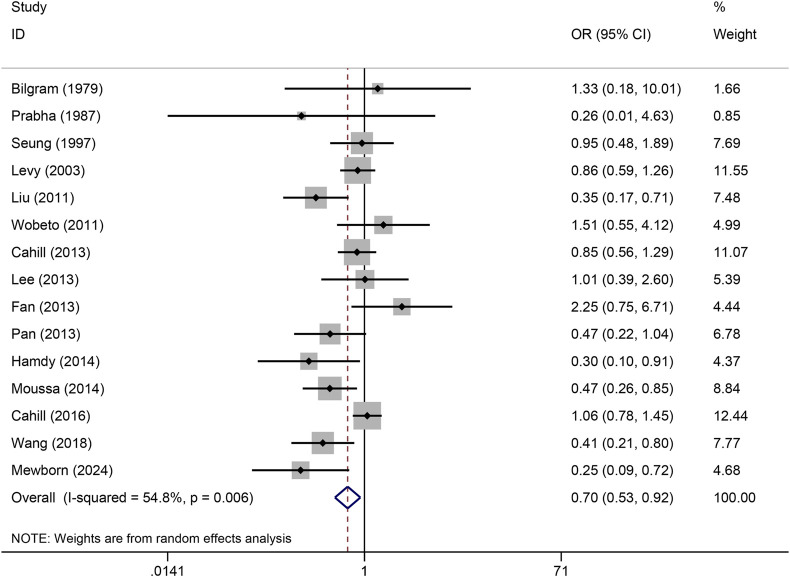
Forest plots of haptoglobin polymorphism and CAD risk under codominant homozygous genetic model.

**FIGURE 5:**
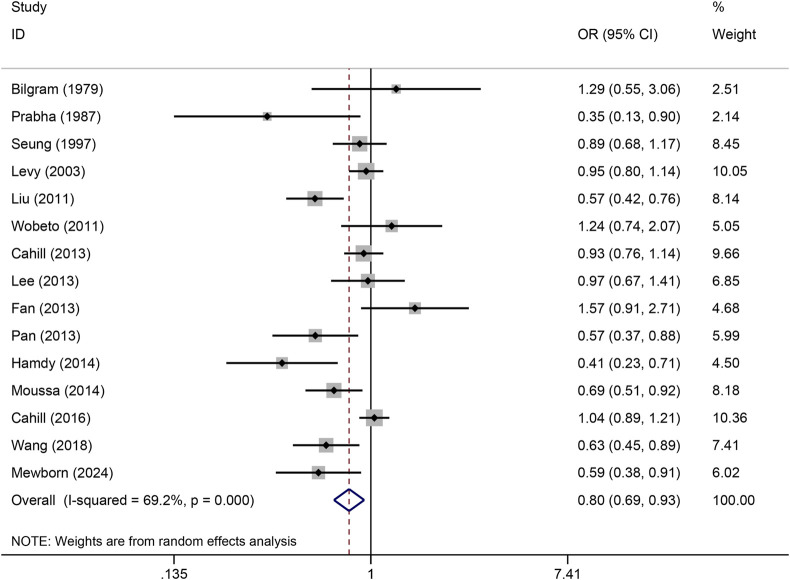
Forest plots of haptoglobin polymorphism and CAD risk under allelic genetic model.

**FIGURE 6:**
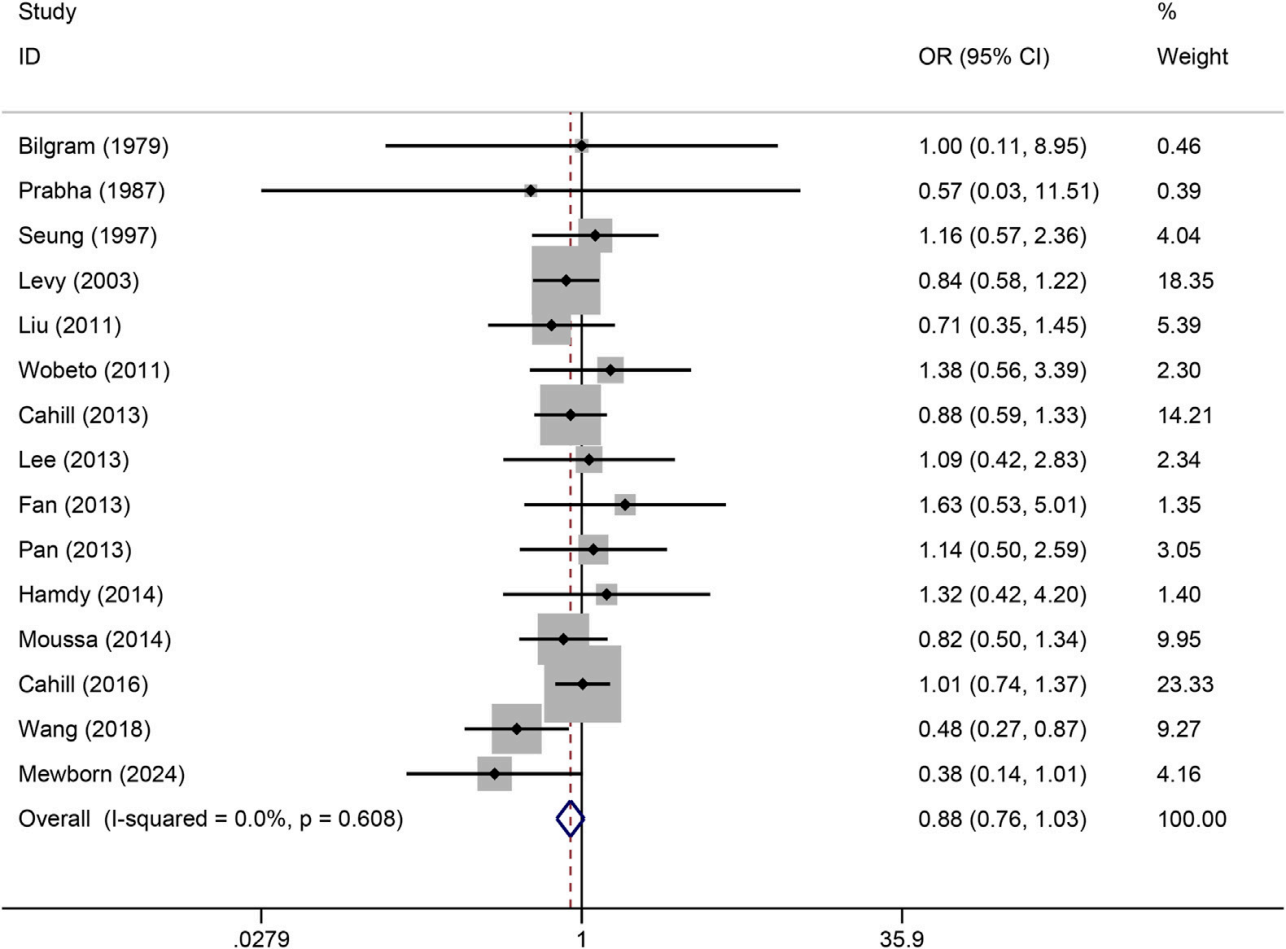
Forest plots of haptoglobin polymorphism and CAD risk under codominant heterozygote genetic model.

**FIGURE 7:**
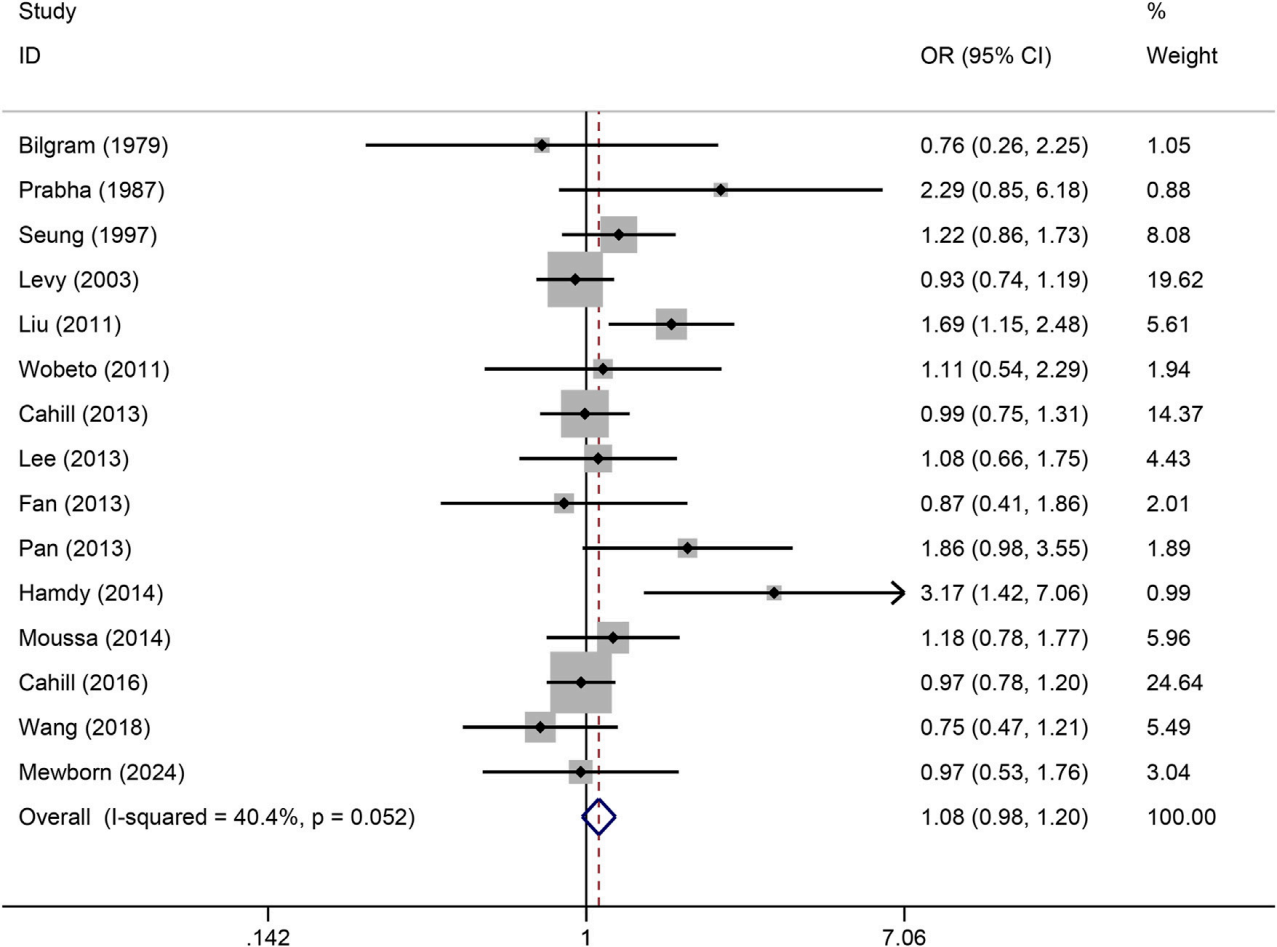
Forest plots of haptoglobin polymorphism and CAD risk under overdominance genetic model.

The pooled association of haptoglobin polymorphism with CAD risk is summarized in Table 2. The combined results showed that a significant association between haptoglobin polymorphism and CAD risk was found under dominant model (OR: 0.82, 95% CI: 0.71–0.95, *p* = 0.007) (Figure 2), recessive model (OR:0.74, 95% CI:0.60–0.92, *p* = 0.005) (Figure 3), codominant homozygote model (OR: 0.70, 95% CI: 0.53–0.92, *p* = 0.010) (Figure 4), allelic genetic model (OR: 0.80, 95% CI: 0.69–0.94, *p* = 0.005) (Figure 5). However, under the codominant heterozygote (Figure 6) and overdominance genetic model (Figure 7), haptoglobin polymorphism did not significantly associated with CAD risk. In addition, we did the subgroup analysis based on ethnicity (Table 2). Results from the subgroup analysis stratified by ethnicity indicated that the haptoglobin polymorphism was associated with CAD susceptibility in the Asian population than that in the Caucasian population. In the Asian subgroup, there was a significant association between haptoglobin polymorphism and CAD risk under recessive (OR: 0.72, 95% CI: 0.52–0.98, *p* = 0.036) and dominant model (OR: 0.70,95% CI: 0.53–0.93, *p* = 0.015), and overdominance genetic model (OR: 1.21,95% CI: 1.03–1.48, *p* = 0.024). In the Caucasian subgroup, no significant association under all genetic model.

**TABLE 2:** Summary of meta-analysis of association of haptoglobin polymorphism and coronary artery disease risk.

Model		Studies	Overall effect			Heterogeneity	
			OR (95% CI)	Z-score	*p*-value	*I* ^2^ (%)	*p*-value
Hp 2-2 vs. Hp 1-1 + Hp 2-1	Overall	15	0.74(0.60, 0.92)	2.78	0.005	67	0.001
	Caucasian	4	0.97(0.83, 1.13)	0.42	0.676	16.2	0.311
	Asian	8	0.72(0.52, 0.98)	2.09	0.036	59.2	0.016
	Egyptian	1	0.25(0.11, 0.54)	3.53	0.001	—	—
	Tunisian	1	0.54(0.33, 0.88)	2.46	0.014	—	—
	Mix	1	1.22(0.54, 2.75)	0.28	0.632	—	—
Hp 2-1 + Hp 2-2 vs. Hp 1-1	Overall	15	0.82(0.71, 0.95)	2.69	0.007	28.3	0.146
	Caucasian	4	0.89(0.74, 1.07)	1.24	0.216	43.7	0.149
	Asian	8	0.70(0.53, 0.93)	2.44	0.015	27.9	0.206
	Egyptian	1	0.59(0.21, 1.67)	0.99	0.321	—	—
	Tunisian	1	0.68(0.43, 1.08)	1.62	0.105	—	—
	Mix	1	1.43(0.62, 3.31)	0.83	0.408	—	-
Hp 2-2 vs. Hp 1-1	Overall	15	0.70(0.53, 0.92)	2.59	0.010	54.8	0.006
	Caucasian	4	0.83(0.59, 1.17)	1.07	0.284	56.2	0.077
	Asian	8	0.67(0.42, 1.060	1.70	0.090	46.9	0.068
	Egyptian	1	0.30(0.10, 0.91)	2.13	0.033	—	—
	Tunisian	1	0.47(0.26, 0.85)	2.50	0.012	—	—
	Mix	1	1.51(0.55, 4.12)	0.80	0.423	—	—
Hp 2-1 vs. Hp 1-1	Overall	15	0.88(0.76, 1.03)	1.60	0.110	0.0	0.608
	Caucasian	4	0.89(0.73, 1.08)	1.21	0.228	0.0	0.300
	Asian	8	0.83(0.62, 1.13)	1.17	0.242	0.0	0.464
	Egyptian	1	1.32(0.42, 4.20)	0.47	0.637	—	—
	Tunisian	1	0.82(0.50, 1.34)	0.81	0.418	—	—
	Mix	1	1.38(0.56, 3.39)	0.70	0.483	—	—
Hp 2 vs. Hp 1	Overall	15	0.80(0.69, 0.94)	2.82	0.005	69.2	0.001
	Caucasian	4	0.93(0.80, 1.08)	0.96	0.338	50.0	0.111
	Asian	8	0.77(0.60, 1.00)	1.95	0.051	65.7	0.005
	Egyptian	1	0.41(0.23, 0.71)	3.13	0.002	—	—
	Tunisian	1	0.69(0.51, 0.92)	2.54	0.011	—	—
	Mix	1	1.24(0.74, 2.07)	0.81	0.419	—	—
Hp 2-1 vs. Hp 1-1 + Hp 2-2	Overall	15	1.08(0.98, 1.20)	1.49	0.137	40.4	0.052
	Caucasian	4	0.96(0.84, 1.10)	0.55	0.584	0	0.991
	Asian	8	1.23(1.03, 1.48)	2.26	0.024	39.4	0.116
	Egyptian	1	3.17(1.42, 7.06)	2.83	0.005	—	—
	Tunisian	1	1.18(0.78, 1.77)	0.79	0.429	—	—
	Mix	1	1.11(0.54, 2.29)	0.28	0.781	—	—

OR, odds ratio; CI, confidence interval.

### Sensitivity analysis and test of heterogeneity

**FIGURE 8:**
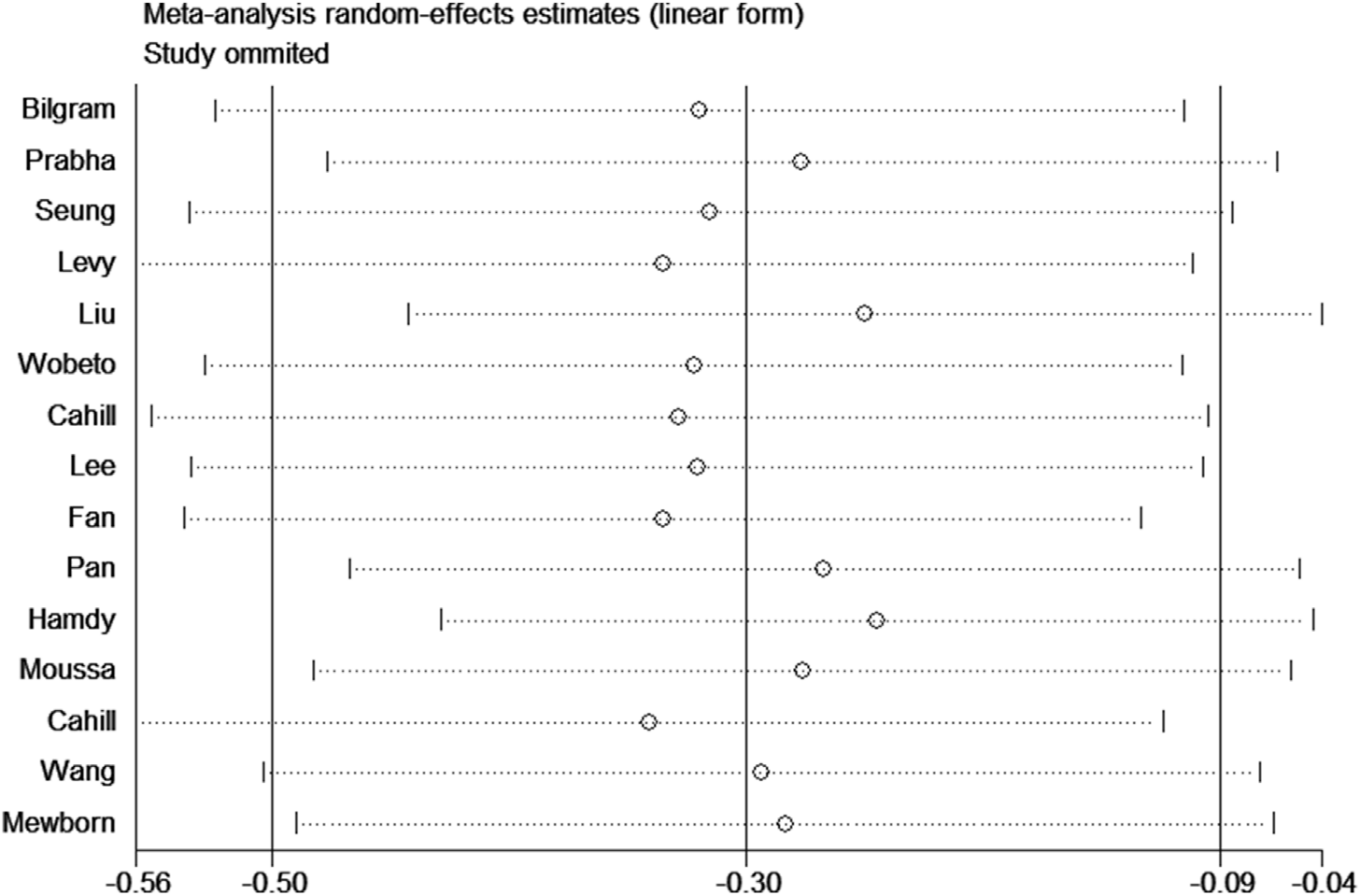
Sensitivity analysis for association between haptoglobin polymorphism and CAD risk under recessive genetic model.

**FIGURE 9:**
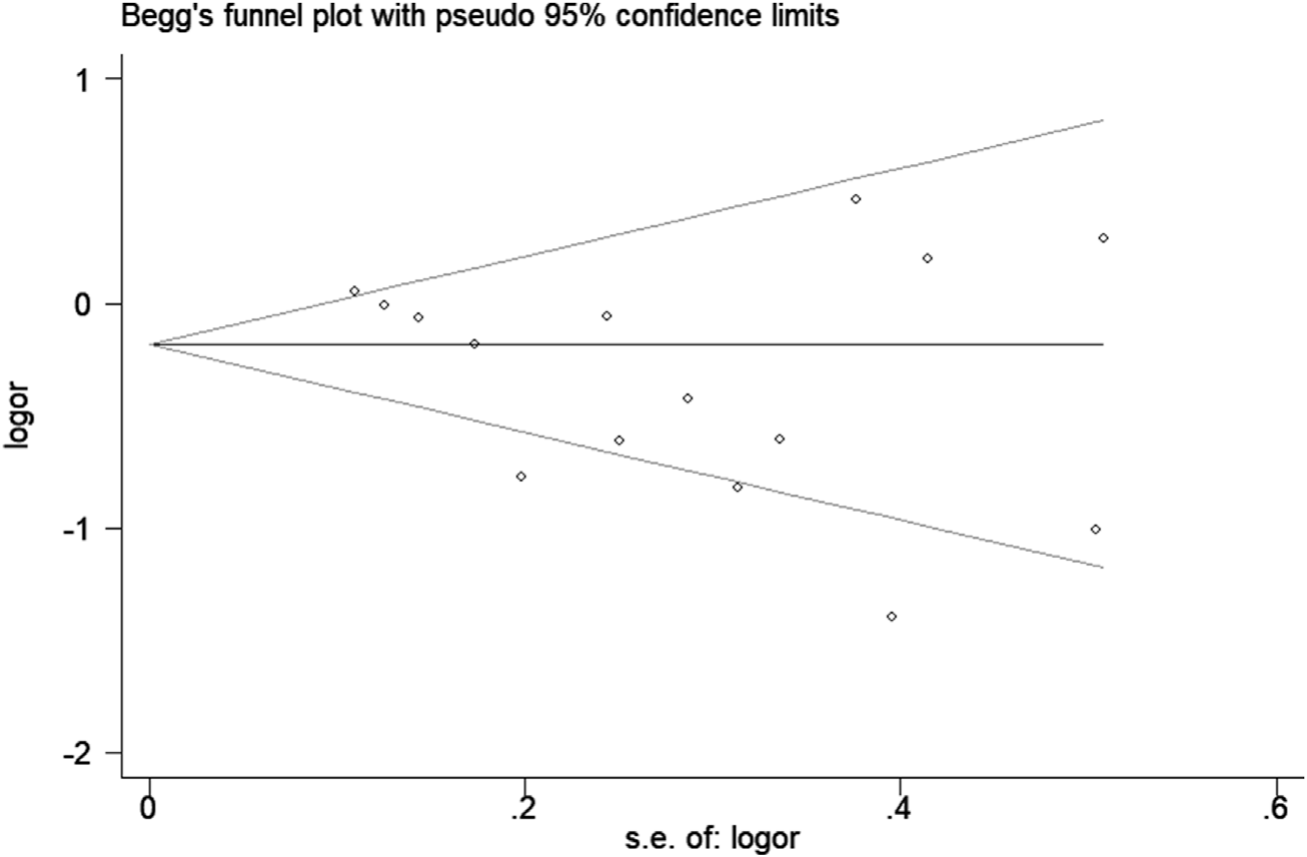
Begg’s funnel plot for association between haptoglobin polymorphism and CAD risk under recessive genetic model.

Sensitivity analysis was used to estimate the effect of individual study on the pooled results (Figure 8). Omitting individual studies did not influenced the pooled results, suggesting that our results were reliable. Begg’s and Egger’s tests were used to evaluate the publication bias (Figure 9). The results showed that there was no publication bias under all genetic models (Table 3).

**TABLE 3:** The effect of publication bias was assessed by Begg’s and Egger’s tests.

Model	Test of Begg (*P*-value)	Test of Egger (*P*-value)
Hp 2-2 vs. Hp 1-1 + Hp 2-1	0.299	0.082
Hp 2-1 + Hp 2-2 vs. Hp 1-1	0.729	0.463
Hp 2-2 vs. Hp 1-1	0.586	0.227
Hp 2-1 vs. Hp 1-1	0.805	0.925
Hp 2 vs. Hp 1	0.216	0.129
Hp 2-1 vs. Hp 1-1 + Hp 2-2	0.255	0.111

## Discussion

This study systematically synthesized the published research on the association between haptoglobin polymorphisms and CAD susceptibility. In the pooled analysis of five different genetic models, we found that individuals with the HP2 genotype had a greater risk of CAD than those with the HP1 allele. Subgroup analysis revealed a significant association between haptoglobin polymorphisms and CAD risk in Asian populations, while in Caucasians, haptoglobin polymorphisms were unrelated to CAD.

Current research indicates that haptoglobin primarily exhibits anti-inflammatory effects (Costacou et al., 2015). When inflammation occurs within the body, haptoglobin synthesis increases dramatically to mitigate further inflammation (Sadrzadeh and Bozorgmehr, 2004). Inflammation plays a crucial role in the development of atherosclerosis and in its association with CAD, which poses a significant threat to human health (Boland and Long, 2021). Haptoglobin binds to free haemoglobin (Hb) to form an HP complex, which effectively reduces the consumption of free Hb by carbon monoxide, thereby lowering the risk of cardiovascular complications and CAD. However, compared to individuals with the HP1-1 genotype, individuals with the HP2-2 genotype have a significantly greater risk of developing CAD. Bacquer et al. reported that haptoglobin 1 increases the risk of CAD and its mortality rate (OR 2.09, 95% CI 1.22–3.60) compared to haptoglobin 2 (De Bacquer et al., 2001). Conversely, Mewborn et al. discovered that patients with the HP2-2 genotype have a fourfold greater incidence of CAD than those with the HP1-1 genotype (OR 3.987, 95% CI 1.39–11.43) (Mewborn et al., 2024). In summary, this meta-analysis suggested that haptoglobin polymorphism was associated with an increased risk of CAD in Asian populations.

Individuals with the HP2-2 phenotype exhibit a reduced clearance rate of macrophage-HP-Hb complexes, which can affect iron deposition, oxidative stress, and the accumulation of active macrophages, all of which are consistent with an increased risk of atherosclerotic cardiovascular diseases (Moreno et al., 2008). A greater proportion of participants with lower Hp concentrations exhibited the HP1-1 phenotype than among those with higher concentrations, which is consistent with the higher affinity of haptoglobin 1-1 for binding to HP (Holme et al., 2009). Given the connection between HP and CAD, patients with the HP1-1 phenotype might have a lower risk of CAD due to their lower plasma HP levels (Lee et al., 2013). Due to impaired clearance of the HP-Hb 2-2 complex, there is an increased concentration of Hp–Hb in Hp 2-2 individuals, which allows the complex to bind to other plasma proteins that are not normally bound, such as high-density lipoproteins (HDL) (Goldenstein et al., 2018). Hp can bind directly to the main apolipoprotein ApoA1 of HDL, and thereby tether Hb to which it is complexed to HDL. This process transforms normal anti-atherosclerotic and antioxidant HDL particles into pro-atherosclerotic pro-oxidative dysfunctional HDL particles (Asleh et al., 2019). In addition, the increased attachment of Hb to HDL may lead to endothelial dysfunction through its interference with HDL function. Firstly, Hb binding to HDL leads to oxidative modification of HDL-associated lipids and proteins, resulting in the inactivation of antioxidant enzymes such as glutathione peroxidase and paraoxonase (Navab et al., 2006). Secondly, Hb can oxidise ApoA1, leading to a significant impairment of its ability to promote cholesterol efflux from macrophages (Salvatore et al., 2007). Thus, the prevalence of microvascular endothelial dysfunction is higher in Hp 2-2 individuals because Hp 2-2 is a poorer antioxidant relative to Hp 1-1, especially for glycosylated Hb (Asleh et al., 2019).

The influence of haptoglobin polymorphisms on CAD risk was obviously nonuniform, which might be prompted by, sample size, racial difference, gender and age. Despite our attempt to conduct a comprehensive meta-analysis of haptoglobin gene polymorphisms and CVD risk, it is essential to consider the limitations of the current studies. First, the subgroup analysis based on race suggested that regional and genetic environmental variations might influence the results. The complexity of gene-environment interactions suggests a need for validation of these findings in diverse racial and ethnic populations. Second, our study focused on the association between haptoglobin polymorphisms and CAD susceptibility in Asians and Caucasians, without examining other populations. Finally, the literature search was primarily in English, potentially overlooking studies in other languages.

In summary, this meta-analysis highlights a significant association between haptoglobin polymorphism and CAD risk in Asian populations. Given its limitations, it is recommended that future studies with larger sample sizes and broader subgroups be conducted to further investigate the relationship between haptoglobin polymorphisms and CAD susceptibility, accounting for the potential influence of genetic and environmental factors.

## Data Availability

The original contributions presented in the study are included in the article/Supplementary Material, further inquiries can be directed to the corresponding author.
