# Supplementary material for: Association between haptoglobin polymorphism and coronary artery disease: a meta-analysis
Source: Front Genet. 2024 Sep 11;15:1434975. doi: 10.3389/fgene.2024.1434975 (PMC11422136; doi:10.3389/fgene.2024.1434975)
Supplement: Supplementary file 2 [file Table2.DOCX]

**Supplementary Table S2. The details of the search strategies.**

|  | |
| --- | --- |
| Database | Search strategy |
| Cochrane Library | ((haptoglobin):ti,ab,kw OR MeSH descriptor: [haptoglobin] in all MeSH products) AND ((polymorphism):ti,ab,kw OR (variant):ti,ab,kw OR (mutation):ti,ab,kw OR MeSH descriptor: [Polymorphism, Genetic] in all MeSH products OR MeSH descriptor: [mutation] in all MeSH products) AND ((coronary artery disease):ti,ab,kw OR (coronary heart disease):ti,ab,kw OR MeSH descriptor: [coronary artery disease] in all MeSH products OR MeSH descriptor: [coronary heart disease] in all MeSH products) |
| PubMed | ("haptoglobins"[MeSH Terms] OR "haptoglobins"[All Fields] OR "haptoglobin"[All Fields]) AND ("polymorphic"[All Fields] OR "polymorphics"[All Fields] OR "polymorphism s"[All Fields] OR "polymorphism, genetic"[MeSH Terms] OR ("polymorphism"[All Fields] AND "genetic"[All Fields]) OR "genetic polymorphism"[All Fields] OR "polymorphism"[All Fields] OR "polymorphisms"[All Fields] OR ("variant"[All Fields] OR "variant s"[All Fields] OR "variants"[All Fields]) OR ("mutate"[All Fields] OR "mutated"[All Fields] OR "mutates"[All Fields] OR "mutating"[All Fields] OR "mutation"[MeSH Terms] OR "mutation"[All Fields] OR "mutations"[All Fields] OR "mutation s"[All Fields] OR "mutational"[All Fields] OR "mutator"[All Fields] OR "mutators"[All Fields])) AND ("coronary artery disease"[MeSH Terms] OR ("coronary"[All Fields] AND "artery"[All Fields] AND "disease"[All Fields]) OR "coronary artery disease"[All Fields] OR ("coronary disease"[MeSH Terms] OR ("coronary"[All Fields] AND "disease"[All Fields]) OR "coronary disease"[All Fields] OR ("coronary"[All Fields] AND "heart"[All Fields] AND "disease"[All Fields]) OR "coronary heart disease"[All Fields])) |
| EMBASE | ('haptoglobin'/exp OR haptoglobin.ti,kw,hw) ANF ('polymorphism'/exp OR polymorphism.ti,kw,hw OR 'variant'/exp OR variant.ti,kw,hw OR 'mutation'/exp OR mutation.ti,kw,hw) AND ('coronary artery disease'/exp OR 'coronary artery disease' OR (coronary AND ('artery'/exp OR artery) AND ('disease'/exp OR disease)) OR 'coronary heart disease'/exp OR 'coronary heart disease' OR (coronary AND ('heart'/exp OR heart) AND ('disease'/exp OR disease))) |
| Web of science | (((TS=(haptoglobin)) AND (TS=(polymorphism) OR TS=(variant) OR TS=(mutation)) AND ((TS= (coronary artery disease) OR TS= (coronary heart disease))) |
| WanFang | (SU=“haptoglobin””) AND (SU=“polymorphism” OR SU=“mutation” OR SU=“variant”) AND (SU=“coronary heart disease” OR SU=“coronary artery disease”) |
