# Supplementary material for: Association between haptoglobin polymorphism and coronary artery disease: a meta-analysis
Source: Front Genet. 2024 Sep 11;15:1434975. doi: 10.3389/fgene.2024.1434975 (PMC11422136; doi:10.3389/fgene.2024.1434975)
Supplement: Supplementary file 3 [file Table3.DOCX]

**Supplementary Table S3. Quality assessments of studies based on Newcastle Ottawa Scale.**

| **First author** | **Is the case definition adequate?** | **Representativeness of the cases** | **Selection of controls** | **Definition of controls.** | **Control for important factors** | **Exposure assessment** | **Same method of ascertainment for cases and controls.** | **Non-response rate** | **NOS score** |
| --- | --- | --- | --- | --- | --- | --- | --- | --- | --- |
| Bilgram | 1 | 0 | 0 | 1 | 1 | 1 | 1 | 1 | 6 |
| Prabha | 1 | 0 | 0 | 1 | 1 | 1 | 1 | 1 | 6 |
| Seung | 1 | 0 | 0 | 1 | 1 | 1 | 1 | 1 | 6 |
| Levy | 1 | 0 | 1 | 1 | 1 | 1 | 1 | 1 | 7 |
| Liu | 1 | 0 | 0 | 1 | 1 | 1 | 1 | 1 | 6 |
| Wobeto | 1 | 0 | 1 | 1 | 2 | 1 | 1 | 1 | 8 |
| Cahill | 1 | 1 | 1 | 1 | 1 | 1 | 1 | 1 | 8 |
| Lee | 1 | 0 | 0 | 1 | 1 | 1 | 1 | 1 | 6 |
| Fan | 1 | 0 | 0 | 1 | 1 | 1 | 1 | 1 | 6 |
| Pan | 1 | 1 | 0 | 1 | 1 | 1 | 1 | 1 | 7 |
| Hamdy | 1 | 0 | 0 | 1 | 1 | 1 | 1 | 1 | 6 |
| Moussa | 1 | 1 | 0 | 1 | 1 | 1 | 1 | 1 | 7 |
| Cahill | 1 | 0 | 1 | 1 | 2 | 1 | 1 | 1 | 8 |
| Wang | 1 | 0 | 0 | 1 | 1 | 1 | 1 | 1 | 6 |
| Mewborn | 1 | 0 | 1 | 1 | 2 | 1 | 1 | 1 | 8 |
